# Niclosamide Blocks Rice Leaf Blight by Inhibiting Biofilm Formation of *Xanthomonas oryzae*

**DOI:** 10.3389/fpls.2018.00408

**Published:** 2018-03-29

**Authors:** Sunil Kumar Sahu, Ping Zheng, Nan Yao

**Affiliations:** State Key Laboratory of Biocontrol, Guangdong Provincial Key Laboratory of Plant Resources, School of Life Sciences, Sun Yat-sen University, Guangzhou, China

**Keywords:** bacterial blight disease, biofilm, extracellular polysaccharide, niclosamide, plant–pathogen interaction, rice, *Xanthomonas oryzae*

## Abstract

Rice (*Oryza sativa*) is the leading source of nutrition for more than half of the world’s population, and by far it is the most important commercial food crop. But, its growth and production are significantly hampered by the bacterial pathogen *Xanthomonas oryzae* pv. *oryzae* (Xoo) which causes leaf blight disease. Earlier studies have reported the antibacterial ability of FDA-approved niclosamide drug against Xoo. However, the underlying mechanism by which niclosamide blocks the growth of Xoo remained elusive. In the present study, by employing the microbiological, microscopical, molecular, bioinformatics and analytical tools we found that niclosamide can directly inhibit the growth of the Xoo by hampering the biofilm formation and the production of xanthomonadin and exopolysaccharide substances (EPS) required for relentless growth and virulence of Xoo. Interestingly, niclosamide was found to specifically suppress the growth of Xoo without affecting other bacteria like *Escherichia coli*. Our electron microscopic observations disclosed that niclosamide disrupts the membrane permeability of Xoo and causes the release of intracellular components. Similarly, the molecular docking analysis disclosed the molecular interaction of niclosamide with the biofilm, virulence and quorum sensing related proteins, which was further substantiated by relative gene expression analysis where niclosamide was found to significantly downregulate the expression of these key regulatory genes. In addition, considerable changes in chemical structures were detected by Fourier Transform Infrared Spectroscopy (FTIR) in response to niclosamide treatment. Overall, our findings advocate the utilization of niclosamide as a safe and potent alternative antibacterial compound to control bacterial blight disease in rice.

## Introduction

More than half of the world’s population relies on rice as a staple source of nourishment. However, over the past decades we have witnessed a serious decline in the overall rice production in several parts of the world due to the unsolicited attack by the several pathogens like fungi and bacteria causing severe diseases, and consequently poses a severe threat to global food security (Niño-Liu et al., 2006; Normile, 2008; Tilman et al., 2017). *Xanthomonas oryzae* pv. *oryzae* (Xoo) is a Gram negative rod-shaped bacterium liable for causing the most devastating bacterial leaf blight disease in rice, which causes annual yield losses of 10–50% and even 100% under severe conditions in many rice growing countries (Niño-Liu et al., 2006; Mansfield et al., 2012; Zhang and Wang, 2013). Wounds or hydathodes are the main entry sites for Xoo where it multiplies in the epitheme, and subsequently enters into the xylem vessels where it starts active multiplication, leading to the initiation of leaf wilting (a typical symptom of the blight disease) (Köplin et al., 1992; Ray et al., 2000).

Xoo is well known to produce a wide varieties of virulence factors, including exopolysaccharides, extracellular enzymes, iron-chelating siderophores, and type III secretion-dependent effectors (Ray et al., 2000; Jha et al., 2007; Liang et al., 2016). The production of large amounts of an exopolysaccharide called xanthan is one of the most important virulence factors of this species. Xanthan is a heteropolysaccharide with a cellulose-like backbone and trisaccharide side-chains of two mannose and one glucuronate residues that are attached to every second glucose moiety of the main chain (Jansson et al., 1975; Köplin et al., 1992). The synthesis of xanthan is regulated by the gumBCDEFGHIJKLM genes, located in a single gene cluster of 12 kb which is expressed as an operon from a promoter upstream of the first gene, gumB (Katzen et al., 1998; Vojnov et al., 1998, 2001). As Xoo is a Gram negative bacterium, it also produces a large amount of lipopolysaccharides (LPSs) in its outer membrane. LPSs are crucial molecules for the viability of Gram negative bacteria and in several phases of host–bacterium interaction such as symbiosis, virulence and tolerance (Silipo et al., 2010; Di Lorenzo et al., 2016). Many studies have demonstrated the role of LPSs in inducing the basal plant defenses thereby entitling LPS molecule as a microbe-associated molecular pattern (MAMP) (Di Lorenzo et al., 2016; Meir et al., 2017). Many plant-pathogenic bacteria use quorum sensing (cell–cell communication) to regulate the expression of factors contributing to virulence (Musthafa et al., 2013; Barel et al., 2015). The quorum sensing (QS), increases the virulence of many species of *Xanthomonas* via the regulation of motility, chemotaxis, stress responses, biofilm dispersal and the synthesis of extracellular enzymes and EPS (LaSarre and Federle, 2013; Shi et al., 2016; Barel et al., 2015).

To control the bacterial leaf blight disease in rice, Bismerthiazole and streptomycin are utilized as the major bactericides, but recent studies have shown that Xoo has developed high resistance against these chemicals (Xu et al., 2010; Zhu et al., 2013; Shi et al., 2015; Yu et al., 2016). Hence, the quest for innovative antibacterial agents remains a major challenge, and such chemical or biological agents are seriously required to control the disease inflicted damages. Over 1 billion people are infected with intestinal nematodes, and many millions are infected with filarial nematodes, flukes, and tapeworms. Niclosamide has been successfully used as an orally bioavailable chlorinated salicylanilide, with anthelmintic and potential antineoplastic activity (Imperi et al., 2013). Niclosamide is a very famous potent drug to cure gastrointestinal tapeworm infections in both humans and animals since 1960s (Weinbach and Garbus, 1969; Kim et al., 2016b). An extensive study in the animal system shows that “niclosamide has effective antiviral activity against the severe acute respiratory syndrome virus, anti-anthrax toxin properties, and anti-neoplastic activity. Niclosamide strongly induces LC3-positive autophagosomes, inhibits the Wnt/ Frizzled pathway, suppresses the autonomous notch-signaling pathway, and inhibits mTOR signaling. Niclosamide uncouples mitochondrial oxidative phosphorylation and thereby slows cell growth” (Weinbach and Garbus, 1969; Wu et al., 2004; Chen et al., 2009; Zhu et al., 2009; Wang et al., 2009; Osada et al., 2011; Fonseca et al., 2012). These findings clearly demonstrate that niclosamide has various curative effects on humans and animals. While searching for a master regulator that effectively functions to protect both animals and plants from infectious diseases recently Kim et al. (2016b) showed that “niclosamide can also inhibit the growth of bacterial plant pathogen Xoo, and it can move long distances from the site of local application to distant rice tissues. Niclosamide also increased the levels of salicylate and induced the expression of defense-related genes thereby suppressing the Xoo-induced leaf wilting. Interestingly, niclosamide had no detrimental effects on vegetative/reproductive growth and yield of rice suggesting that niclosamide can be effectively used to block bacterial leaf blight in rice without any negative effects.” However, in their study the underlying mechanism of action of niclosamide on *Xanthomonas oryzae* pv. *oryzae* remained unexplored. This intrigued us to further explore and establish the molecular mechanisms by which niclosamide inhibits Xoo’s bacterial growth.

## Materials and Methods

### Bacterial Strains and Culture Conditions

The *Xanthomonas oryzae* pv. *oryzae* pathogens were obtained from the Institute of Crop Protection, Guangzhou, China. PXO99 and GDIV strain of Xoo were grown in nutrient broth (NB) medium in conical flasks or 50 ml falcon tubes in a shaking incubator set at 28 ± 1°C (Zhu et al., 2013). Nutrient Agar (NA) medium was prepared with “1 g of yeast extract, 3 g of beef extract, 5 g of polypeptone, 10 g of sucrose, and 15 g of agar powder per 1000 mL of distilled water (pH 7.0–7.2). NB medium contained the same components but lacked agar powder” (Shi et al., 2015). Until otherwise stated PXO99 and *E. coli* strain DH5α were used for all the experiments.

### Plant Materials, Growth Conditions, Chemicals and Pathogen Infestation

Rice (*Oryza sativa* ‘Nipponbare’, japonica variety) obtained from Shandong Academy of Agricultural Sciences, Guangzhou was surface sterilized by agitation in 2% sodium hypochlorite for 20 min, followed by washing thrice in demineralized water. The seeds were overnight soaked in distilled water, and were germinated for 2 days at 28°C on wet filter paper, and then the seeds were transplanted into 96-well plates to grow in hydroponic solution for 1 week. Finally, the seedlings were propagated in the greenhouse (28 ± 1°C, 70% relative humidity and 10/14 light regimen). For stock solutions, niclosamide (Sigma-Aldrich, Product ID: 36177) was dissolved in dimethyl sulfoxide (DMSO) (Sigma-Aldrich, Product ID: D5879) at a concentration of 5 mg/ml. The stock solutions were diluted in distilled water immediately before use. The fully expanded 4th and 5th leaves staged rice seedlings were inoculated by Xoo bacterial suspensions (OD_600_ = 0.5) by leaf-tip-clipping method (Kauffman et al., 1973). The rice plants were sprayed with 10 µg/ml niclosamide at 4-day intervals (Kim et al., 2016b). Plants inoculated with the 0.02% Tween-20 solution were used as mock-inoculated controls. The distance from the tip to the leading edge of grayish to chlorotic tissue was taken as a measure of the progression of blight disease. Disease symptoms were monitored and recorded at regular intervals.

### *In Vitro* Antibacterial Activity

In this study, niclosamide was assessed for antibacterial activities against Xoo via *in vitro* turbidimeter test (Dalgaard et al., 1994). Briefly, “Dimethylsulfoxide (DMSO) in sterile distilled water served as an untreated blank control. Approximately 40 µL of solvent NB containing Xoo, incubated on the phase of logarithmic growth, was added to 5 mL of solvent NB containing the test compounds. The inoculated test tubes were incubated at 28°C and continuously shaken at 180 rpm for 24–48 h until the bacteria were incubated on the logarithmic growth phase. The growth of the cultures was monitored by a Synergy H1 detector (BioTek, Winooski, VT, United States) by measuring the optical density at 595 nm (OD_595_). The inhibition rate “I” was calculated by the following formula:

$$\begin{array}{c} \mathrm{Inhibition}\;\mathrm{rate}\;I\;(\%)\;=\;(C\;-\;T)/C\;\times \;100 \end{array}$$

where C is the corrected turbidity values of bacterial growth on untreated NB (blank control), T is the corrected turbidity values of bacterial growth on treated NB, and I represents the inhibition rate” (Shi et al., 2015).

### Biofilm Assay

Biofilm formation in glass test tube was quantified as described previously (Pratt and Kolter, 1998; Wang et al., 2014). Briefly, “the bacteria were grown in NB with shaking to the mid-exponential growth phase and then diluted to 1:100 in fresh NB. About 2 mL of a diluted bacterial suspension was placed in each glass tube with 5 µg/mL niclosamide and incubated at 28°C for 72 h. The culture medium was poured out, and attached bacterial cells were gently washed three times with distilled water. The cells were then stained with 0.1% crystal violet (2 mL) for 15 min. Unbound crystal violet was poured out, and the glass tubes were washed three times with water. The crystal violet-stained cells were solubilized in DMSO (2 mL). Biofilm formation was quantified by measuring the absorbance at 570 nm using a Synergy H1 detector (BioTek, Winooski, VT, United States). Three replicates were used for quantitative measurement.”

### Confocal Laser-Scanning Microscopic (CLSM) Analysis of Biofilms

Coverslips (18 mm) were first sterilized by soaking in absolute ethanol followed by drying under the flame and then placing in well of a 6-well plate. 3 mL of log phase grown culture of Xoo and *E. coli* then added into 6 well plates containing the sterile coverslips and were treated with or without 5 µg/mL niclosamide and incubated under stationary conditions for 4 days. Prior to confocal observation, the medium was carefully discarded and planktonic bacteria were carefully washed out with 100 mM PBS (pH = 7.4). Coverslips containing biofilm were then carefully removed from the well and stained with fluorescein diacetate (FDA) (F7378, Sigma-Aldrich, United States) at the concentration of 50 µg/mL and imaged using a confocal laser-scanning microscope (Zeiss LSM 880, Germany).

### Quantitative Determination of EPS Production

Bacterial cells were grown in NB supplemented with different concentrations (20, 10, 5, and 2.5 µg/mL) of niclosamide to measure the effect of EPS production of Xoo in culture supernatants at 28°C for 72 h. Subsequently, “10 mL portions of the cultures were collected, and the cells were removed by centrifugation at 8000 *g* for 20 min (Guo et al., 2010). Finally, three volumes of ethyl alcohol were added to the supernatants. The precipitated EPS were pelleted via centrifugation, dried, and weighed. The test was performed three times independently” (Vojnov et al., 1998).

### Quantification of Xanthomonadin

Measurement of xanthomonadin pigment was done as mentioned in the method illustrated by Wang et al. (2015). Briefly, “the Xoo cells collected by centrifuging 4 mL broth suspension with and without niclosamide was mixed with 1 mL 100% methanol. The mixtures were further incubated in darkness for 10 min kept on rotating shaker followed by centrifugation at 12,000 *g* for 8 min to collect the supernatant. The xanthomonadin pigment was estimated by measuring the absorbance at OD445 and the result was denoted relative to the cell density measured before the assay (OD595).”

### Cell Viability and Superoxide Radical ($O_{2}^{•–}$) Assay

The viability of Xoo cell and hypothetical possibility of superoxide radical anion ($O_{2}^{•–}$) production was evaluated by monitoring the absorption of XTT (2,3-bis (2- methoxy-4-nitro-5-sulfophenyl)-2H-tetrazolium-5-carboxanilide) (X4626, Sigma-Aldrich, United States) (Rasool et al., 2016). Superoxide radical anion ($O_{2}^{•–}$) reduces XTT to form water-soluble XTT-formazan with the maximum absorption at 470 nm. Bacterial dispersions treated with niclosamide were mixed with 1 mL of 0.4 mM XTT. The mixture was incubated in dark for 5 h; afterward, the changes in absorbance at 470 nm were monitored with Synergy H1 hybrid multi-mode reader (Biotek, United States).

### Electron Microscopy

Both scanning electron microscopy (SEM) and transmission electron microscopy (TEM) analysis were used to determine the effects of niclosamide on Xanthomonas cells at the ultrastructural level. Xoo and *E. coli* grown up to log phase were treated with 5 µg/ml of niclosamide for 12 h. Untreated cells were used as a control. After centrifugation at 4000 *g* for 10 min, the cells were collected and washed thrice with 100 mM phosphate buffer saline (PBS) to remove unsolicited media and other components (Shi et al., 2016). “Then the pellet was overnight prefixed with 2.5% glutaraldehyde at 4°C. Fixed cells were rinsed three times for 10 min with 100 mM PBS, post-fixed for 3 h in 1% osmium tetroxide, and dehydrated through an ethanol gradient followed by critical point drying. For SEM analysis, samples were coated with gold and analyzed on a Hitachi S-3000N scanning electron microscope (Hitachi, Japan). For TEM analysis, samples were embedded in Epon 812, sectioned with an ultramicrotome and examined under a JEOL JEM-100CX-II transmission electron microscope.”

### Molecular Modeling and Docking

The protein sequences of XanA (Accession number: ACD61040), GumB (Accession number: ACD59988) and RpfB (Accession number: AAL06344) were used as input in Phyre2 server (Kelley et al., 2015). An intensive mode was used for modeling where the server attempts to create a complete full-length model of a sequence through a combination of multiple template modeling and simplified *ab initio* folding simulation. Next, the chemical structures of niclosamide, bismerthiazol and probenazole were generated from the SMILES notation (Simplified Molecular Input Line Entry Specification) in the Chemsketch Software^1^. The active sites of each protein were predicted by COFACTOR (Zhang et al., 2017). To explore the protein-ligand interactions, the molecular docking of niclosamide was carried out in Argus Lab 4.0.1 software (Sahu et al., 2012). Docking simulations were performed by selecting “GA Dock” as the docking engine. Grid resolution was set to 0.40 A°. Calculation type was set to “Dock” mode, and “flexible mode” was selected for the ligand. The docked structure was saved as “.pdb” file and molecular interaction between ligand and the target protein was visualized using Discovery Studio 2017 R2.

### RNA Extraction, cDNA Synthesis, and RT-qPCR Analysis

RNA Extraction, cDNA Synthesis, and RT-qPCR Analysis were peformed according to descriptions given by Shi et al. (2015) and Singh et al. (2017). Briefly, “the bacteria were grown in NB medium at 28°C with shaking at 180 rpm, and 3 mL samples of Xoo strain cultures were collected at 12 h after bacterial cells were incubated with niclosamide. Bacterial cells were centrifuged at 5000 *g* for 10 min, and the total RNA was extracted using E.Z.N.A. bacterial RNA kit (R6950-00, Omega Bio-tek) and RNA purity was evaluated using a NanoDrop ND-1000 spectrophotometer (NanoDrop Technologies, Wilmington, DE, United States). All OD260/OD280 values of RNA were between 1.8 and 2.2. For each sample, 1 µg RNA was reverse transcribed into cDNA using Prime ScriptR^®^ RT reagent kit (TAKARA, DRR047A). Real-time PCR was performed with SYBR Premix Ex Taq kit (Takara, RR820L) according to the manufacturer’s instructions, and quantitatively analyzed by Step One PlusTM real-time PCR systems (ABI). The PCR cycle consisted of the following steps: 30 s at 95°C and 40 cycles of 20 s at 95°C and 30 s at 59°C. The 2^-ΔΔ^CT method (59) was used to determine the relative expression level of target genes according to the expression level of gyrB (the endogenous control). All the experiments were performed in triplicates.” The list of primers used in the present study is listed in **Supplementary Table S1**.

### Fourier Transform Infrared Spectroscopic (FT-IR) Analysis

Xoo and *E. coli* grown up to log phase were treated with 5 µg/ml of niclosamide for 12 h. The untreated cells were used as a control. After incubation, the cell suspensions were collected by centrifuging at 5000 *g* for 10 min and the supernatants were discarded. The cell pellets were freeze-dried for 16 h under vacuum (Sentry 2.0, SP Scientific, United States). The disks for FTIR analysis were prepared by pressing a mixture of 1 mg freeze-dried bacterial powder along with 100 mg of potassium bromide (KBr) powder in a hydraulic press (Rama Devi et al., 2016). The FT-IR spectra of niclosamide treated and untreated samples were recorded at the range of 4,000–400 cm^-1^ using Nicolet 6700 Thermo Scientific spectrophotometer, and the variations in the components of cell membrane were determined using OMNIC software 8.2 (Thermo Fisher Scientific Inc.) were analyzed by subtracting the spectrum of pure KBr. For each spectrum, 16 scans were collected at a resolution of 4 cm^-1^. Each sample was scanned with three different pellets under identical conditions. The average of three replicates was used to analyze the spectra using ORIGIN 6.0 software (OriginLab Corporation, Northampton, MA, United States).

### DNA Extraction and DNA Cleavage Assay

DNA was extracted using E.Z.N.A. Bacterial DNA kit (D3350-00, Omega Bio-tek) and DNA purity was evaluated using a NanoDrop ND-1000 spectrophotometer (NanoDrop Technologies, Wilmington, DE, United States). The OD260/OD280 values of DNA were between 1.8 and 2.0. The extracted DNA (5 µl) was mixed with 5 µl of 5 µg/mL or 5 mg/mL niclosamide and incubated at 28°C for 30 m. Untreated DNA (5 µl) was used as a control. After incubation, the samples were run on a 1.0% agarose gel buffered with 1X TAE at 130 V for 20 min.

### Statistical Analysis

Each experiment was performed in three technical replicates and three biological replicates. Mean significant values were determined by Student’s *t*-test using SPSS package (SPSS V16.0, SPSS, Inc., Chicago, IL, United States). In each statistical data values are means ± SD from triplicate biological repeats. Significant differences were determined by analyzing the comparison between the control and the treated samples using the Post Hoc Test (^∗^*p* < 0.05 and ^∗∗^*p* < 0.01).

## Results

### Effect of Niclosamide on the Growth of Xoo

**FIGURE 1:**
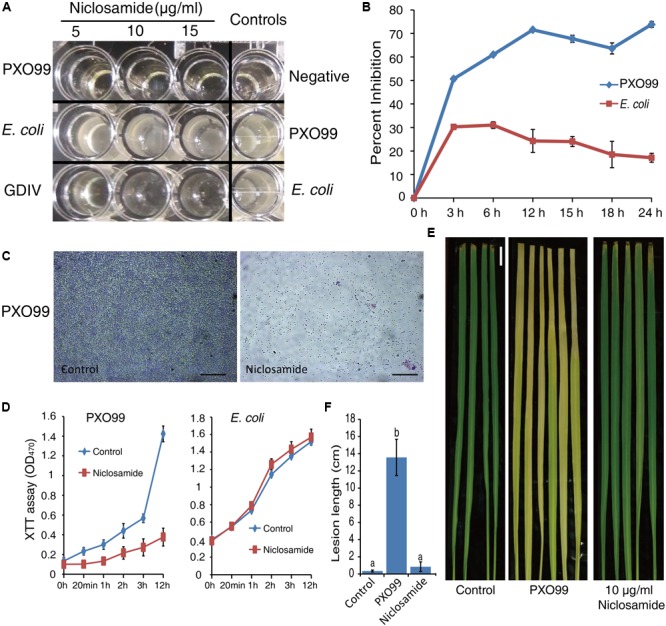
Niclosamide perturbs the growth of *Xanthomonas oryzae* pv. *oryzae* (Xoo) and blocks the progression of bacterial blight disease in rice. **(A,B)** Inhibition rate of niclosamide against Xoo. Firstly, the Xoo was grown up to the logarithmic phase in nutrient medium (NM), and then it was treated with increasing concentration of niclosamide and incubated at 28°C at 180 rpm in shaking incubator for 24–48 h and the growth of the cultures were monitored on a microplate reader by measuring the optical density at 595 nm. Results are means of three technical replicates and the bar indicates the standard deviation. The experiment was repeated three times with similar results. **(C)** Microscopic observation of crystal violet stained Xoo grown in the presence or absence of 5 µg/ml niclosamide. (40X, scale bar = 100 µm). **(D)** Assessment of cell viability and ROS production by XTT assay. The log phase grown bacterial dispersions were treated with 5 µg/ml niclosamide and immediately mixed with 1 mL of 0.4 mM XTT. The changes in absorbance at 470 nm were monitored with a microplate reader at the indicated time points. **(E,F)** Significant reduction in bacterial blight disease by niclosamide. The fully expanded 4th and 5th leaves staged rice seedlings were inoculated by Xoo bacterial suspensions (OD600 = 0.5) by leaf-tip-clipping method. The rice plants were sprayed with 10 µg/ml niclosamide at 4-day intervals (Kim et al., 2016b). Plants inoculated with the 0.02% Tween-20 solution were used as mock-inoculated controls. The lesion length was measured after 14 days of inoculation. Values are means ± SD from triplicate biological repeats. Significant differences were determined by a Post Hoc Test (*p* < 0.05) using different letters. Bar = 1 cm.

According to the earlier reports (Kim et al., 2016a,b), we first confirmed the antibacterial activity of niclosamide against two pathogenic *Xanthomonas oryzae* pv. *oryzae* strains PXO99 and GDIV corresponding to race 6 and race 4 respectively. In addition, we also tested the efficacy of niclosamide on Xoo by the turbidity assay on a microtitre plate. As anticipated we found a strong inhibitory (>80%) potential against both the strains of Xoo at the concentration of 5, 10, and 15 µg/ml niclosamide, evident by the clear and transparent well similar to the negative control (only NAM medium) (**Figures 1A,B**). However, no such inhibition was observed against *E. coli* (opaque and turbid wells) indicating that niclosamide specifically blocks the growth of Xoo (**Figures 1A,B**). This observation was further confirmed by crystal violet staining of a 24 h grown Xoo colony on a glass slide which clearly showed a considerable reduction in the number of Xoo cells under the light microscope (**Figure 1C**). Further by employing the XTT assay we measured the ROS production and the viability of Xoo in the presence of niclosamide drug which clearly showed a significant reduction in the viable Xoo in comparison to untreated controls. However, no significant changes were observed in *E. coli*’s viability (**Figure 1D**). After ascertaining the strong inhibitory activity of niclosamide against Xoo, we further performed an in *vivo* bioassay directly on the rice plants. Interestingly, at the concentration of 10 µg/ml niclosamide impeded the development of bacterial blight disease in rice leaves. The plants sprayed with niclosamide were as green and lesion free as that of the untreated plants (**Figure 1E**), and hence there was a more obvious significant reduction in the lesion length compared with control (**Figure 1F**). Therefore, these data clearly demonstrate that niclosamide has a very strong antibacterial activity in both *in vitro* and *in vivo* conditions and can be effectively used as an alternative strategy to control the most devastating bacterial blight disease in the major crop plant rice.

### Niclosamide Perturbs the Biofilm Formation by Xoo

**FIGURE 2:**
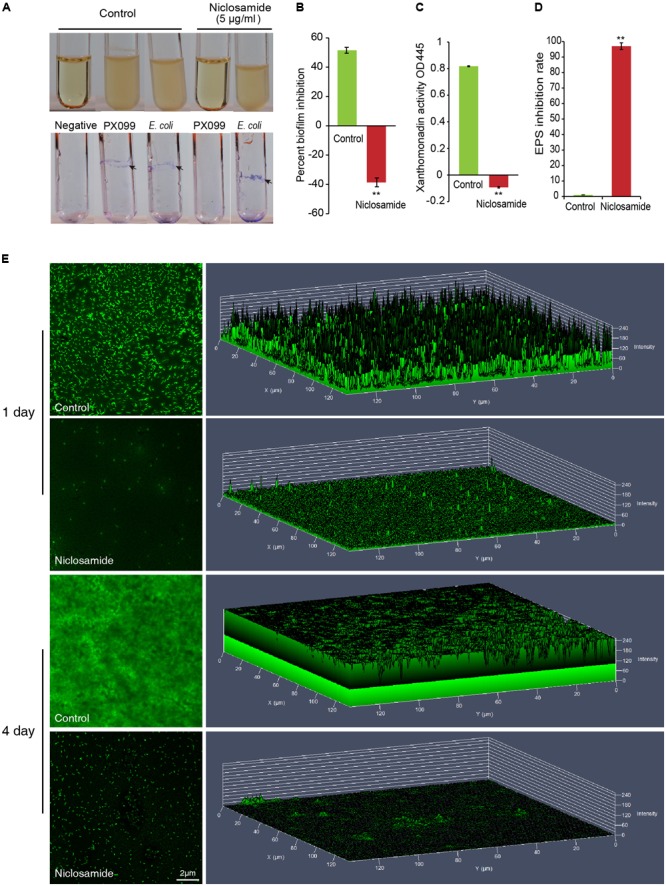
Niclosamide restricts the developmental of biofilm in Xoo. **(A)** Upper panel shows the growth of bacteria (Xoo and *E. coli*) in nutrient agar medium after 72 h of incubation in the presence or absence of 5 µg/ml niclosamide. Turbid and opaque tubes indicate bacterial growth while translucent ones represent the growth inhibition. The bottom panel depicts the crystal violet stained adherent biofilms on glass test tubes. **(B)** The percentage of biofilm inhibition by niclosamide drug. **(C)** Reduction in xanthomonadin activity after niclosamide treatment. **(D)** Inhibition rate of exopolysaccharide substance (EPS) production by niclosamide. **(E)** Biofilm formation by Xoo is hampered by Niclosamide. Xoo were grown on cover slides with or without 5 µg/ml niclosamide, and were stained with fluorescein diacetate (FDA) prior to observation under confocal laser scanning microscopy. The left panel shows the Xoo cells while right panel shows the 2.5D stacked image of biofilm growth at the indicated time points. The height represents the FDA signal intensity representing the bacterial density and biofilm thickness. Scale bar = 2 µm. All the experiments were repeated thrice using independent samples with similar results. Values are means ± SD from triplicate biological repeats. Significant differences were determined by a Post Hoc Test (^∗^*p* < 0.05 and ^∗∗^*p* < 0.01).

In order to gain further insight into the inhibitory mechanism of niclosamide, we utilized the crystal violet assay to monitor the formation of biofilm by Xoo (**Figure 2A**). As expected niclosamide was found to restrict the developmental of biofilm by Xoo. The upper panel in **Figure 2A** shows the growth of bacteria (Xoo and *E. coli*) in nutrient agar medium after 72 h of incubation in the presence and absence of 5 µg/ml niclosamide. Turbid and opaque tubes indicate bacterial growth while translucent ones represent the growth inhibition. The bottom panel depicts the crystal violet stained adherent biofilms on glass test tubes. Quantitative estimation by microtitre plate reading showed >40% inhibition of biofilm (**Figure 2B**). Then we also evaluated if niclosamide has any effect on the xanthomonadin and EPS activity of Xoo which is essential for the growth and development of biofilm. Remarkably the niclosamide drug was found to significantly block the production of Xanthomonadin (**Figure 2C**) and EPS production (**Figure 2D**). Then to better understand the inhibitory action of niclosamide on biofilm we employed the confocal laser scanning microscopy (CLSM) to visualize the growth of biofilm at different stages of development. Xoo was grown on cover slides with or without 5 µg/ml niclosamide, and were stained with fluorescein diacetate (FDA) and the fluorescence was immediately observed under the confocal microscope. Notably, a substantial reduction in the growth of biofilm was observed after 24 h of treatment in comparison to the untreated control (**Figure 2E**). The 2.5D stacked image of biofilm growth shows a strong signal intensity (at the right panel of **Figure 2E**) indicating the development of biofilm and bacterial density. Consistent with our above assay there was an extensive reduction in biofilm formation by Xoo even after 4 days of treatment, while the control showed a continuous and noteworthy formation of biofilm indicated by the higher FDA signal intensity and thicker 2.5D image (**Figure 2E**). Collectively these results reveal that niclosamide diminishes the xanthomonadin and extracellular polysaccharides (EPS) synthesis, thereby constraining the development of biofilm in Xoo.

### Niclosamide Induces Morphological and Ultrastructural Changes in Xoo

**FIGURE 3:**
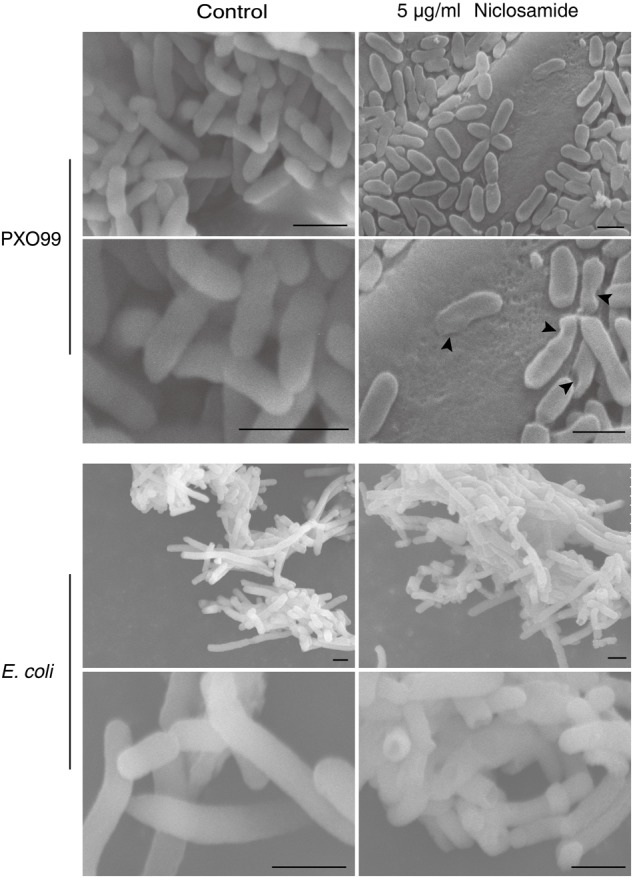
Niclosamide causes ultrastructural modifications in Xoo. Scanning electron microphotographs of Xoo treated with sterile distilled water (control) and with 5 µg/ml niclosamide. Black arrow indicates the damage caused by niclosamide drug. Bar = 1 µm.

**FIGURE 4:**
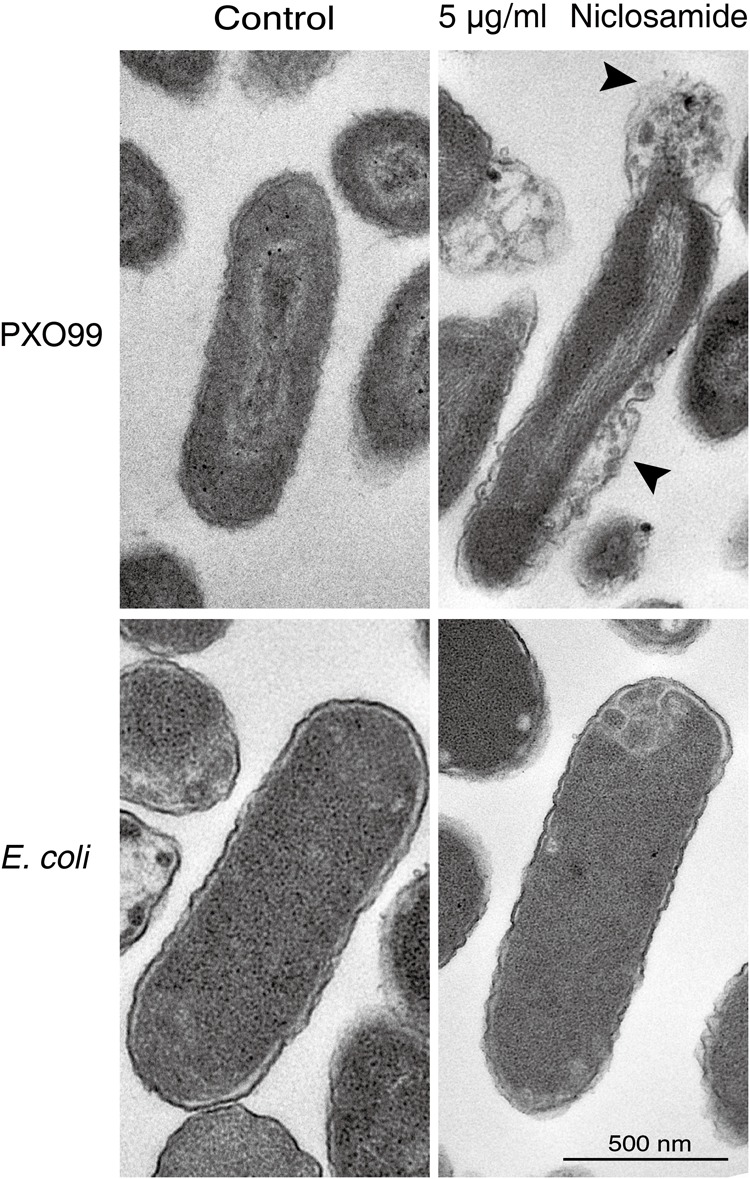
Niclosamide disrupts the membrane permeability of Xoo, and causes the release of intracellular components. Transmission electron microphotographs of Xoo treated with 5 µg/ml niclosamide or sterile distilled water (control). Bar = 500 nm.

The morphological changes and the membrane integrity of Xoo and *E. coli* in response to niclosamide treatment were further evaluated by scanning and transmission electron microscopy. The scanning electron micrograph revealed that normal untreated cells of both Xoo and *E. coli* were typically rod shaped with a normal, smooth and bright surface without any apparent cellular debris (**Figure 3**). However, niclosamide treated Xoo showed irregular shape with sunken surfaces, while *E. coli* cells were the same as the control without any significant damage (**Figure 3**). TEM observations indicated that the Xoo cell membranes were heavily disrupted with noticeable irregular shape and morphology (**Figure 4**). Serious structural changes were also evidenced by the presence of a large amount of debris and distinct formation of potholes on the surface. The cell wall disruption instigated the leakage of the intracellular bacterial content. However, the *E. coli* cells remained intact similar to the SEM observations (**Figure 4**). Thus by the electron microscopy, we concluded that niclosamide disrupts the membrane integrity and causes the release of bacterial content, which eventually leads to the inhibition of Xoo’s growth.

### Molecular Docking Reveals the Inhibition of Xanthan, EPS and Diffusible Signal Factor (DSF) Family Proteins by Niclosamide

**FIGURE 5:**
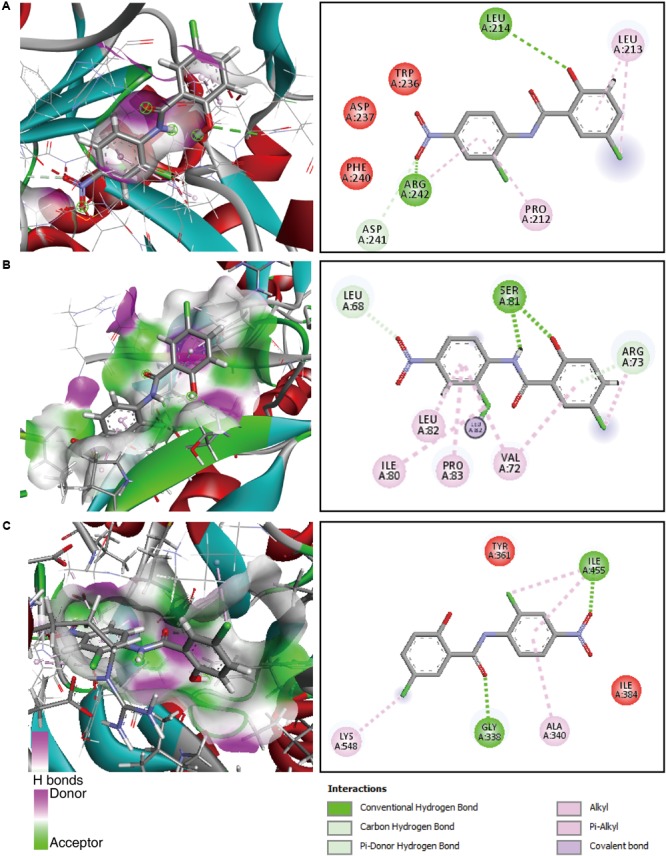
Molecular docking reveals the physical interaction of niclosamide with the biofilm and quorum sensing related proteins **(A)** GumB, **(B)** RpfB 3D and **(C)** XanA. Left panel shows the 3D interaction pose of niclosamide in the active site of the respective proteins. While, right panel shows the 2D view of intermolecular H bonding interaction and neighboring residues.

Our above results indicated a strong inhibition of xanthan and EPS production by niclosamide drug. Therefore, in order to further understand the molecular interactions between niclosamide and the proteins responsible for the production xanthan and EPS we performed the molecular docking analyses. As the crystal structures of the target protein (XanA, GumB and RpfB) are not characterized yet, we first did the homology modeling by using the Phyre2 server. For XanA and RpfB, 100% of residues were modeled at >90% confidence. However, for gumB only 78% of residues were modeled at >90% confidence. Then the ligand binding site was predicted by using COFACTOR and the proteins were subjected to molecular docking analysis in Argus Lab software. The results showed a higher binding affinity of niclosamide with all the tested proteins compared to the controls bismerthiazol and probenazole. For instance, the binding affinity of niclosamide was > -8 Kcal/mol with XanA, GumB and RpfB, while for control it was below -7 Kcal/mol, exhibiting the strong interaction of niclosamide with the proteins (**Table 1**). Then to understand the interaction between the docked protein and ligand, the structure was further visualized in Discovery Studio. Niclosamide was found to form two hydrogen bonds (dashed green line) with Leu214 and Arg242 of XanA protein. In addition, Asp241 formed a pi-donor hydrogen bond (dashed light blue line). The structure was further stabilized by the pi-alkyl bond (dashed pink line) of Leu214 and Pro212 amino acids (**Figure 5A**). Similarly, niclosamide formed two hydrogen bonds with Ser81 and one hydrogen bond with Leu82. Moreover, Arg73 also formed a pi-donor hydrogen bond with niclosamide. The structure was further supported by several alkyl and pi-alkyl bonds indicated by the dotted pink arrow (**Figure 5B**). The molecular interactions of the positive controls are shown in **Supplementary Figure S1**. Gly338 and Ile445 interacting residues of the RpfB protein formed two hydrogen bonds with niclosamide. Ile445 also formed a pi-alkyl bond along with Lys548 and Ala340 (**Figure 5C**). Collectively, these results suggest that there is a strong binding affinity of niclosamide with the target proteins implying that niclosamide has the potential to block the activity of these proteins.

**Table 1:** The binding affinity energies of niclosamide and other commercial drugs against the target proteins.

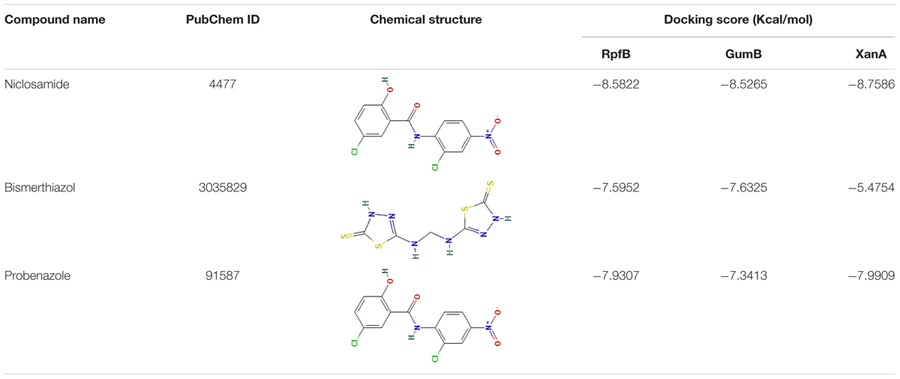

### Niclosamide Downregulates the Expression of Xanthan, EPS and Diffusible Signal Factor (DSF) Related Genes

**FIGURE 6:**
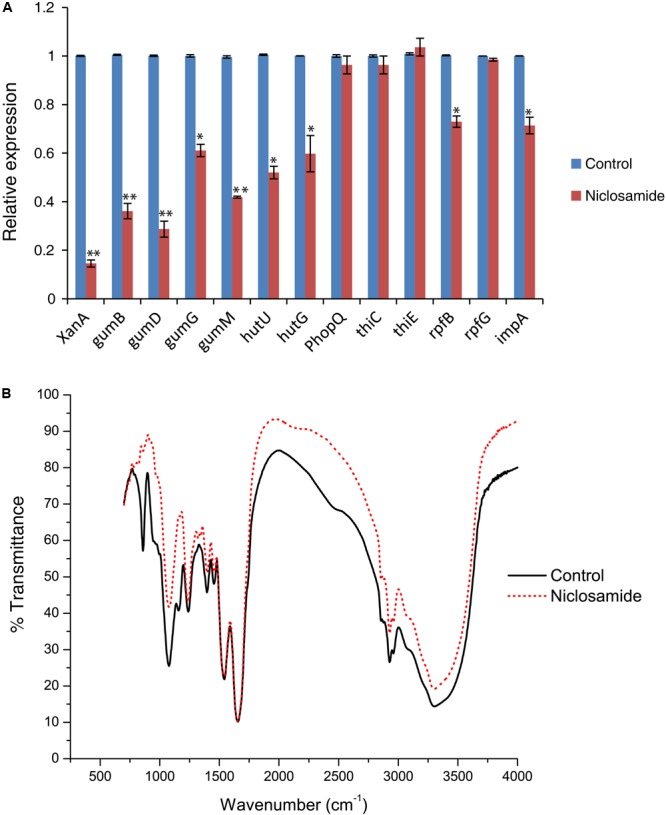
Niclosamide downregulates the expression of biofilm, EPS and quorum sensing related genes, and causes substantial changes in chemical structures of Xoo. **(A)** qRT-PCR analysis showing the differential gene expression in Xoo in the presence or absence of 5 µg/ml niclosamide. Only “Xoo” served as control while “Xoo+ niclosamide” represents the treatment in the figure. gyrB was used as an endogenous control. Gene expression values are presented relative to untreated Xoo (set as 1). Results are means of three technical replicates and the bar indicates the standard deviation. Asterisk indicates ^∗∗^*P* < 0.01 and ^∗^*P* < 0.05. **(B)** Fourier transform infrared spectroscopy (FTIR) showing the chemical modifications in Xoo after 12 h of niclosamide (5 µg/ml) treatment. Dashed red line indicates the shift in the IR spectra compared to untreated samples (solid black line).

Our bioinformatics analyses predicted the physical interaction of niclosamide with the biofilm and quorum sensing related proteins. To prove this hypothesis and gain further insight into the molecular mechanism of niclosamide, qRT PCR analysis was carried out. As expected a significant reduction in the expression of gum, xanthan and EPS genes were observed. XanA gene which is involved in the production of xanthan and EPS biosynthesis showed a significant 5.5 fold reduction in its expression compared to the control (**Figure 6A**). Similarly, all the tested genes (gumB, gumD. gumG, and gumM) corresponding to gum gene cluster responsible for EPS biosynthesis were also downregulated by 3.5, 4, 1.6, and 2.5 fold respectively (**Figure 6A**). Moreover, the expression of impA (gene coding for an inner membrane protein), and rpfB responsible for the production of diffusible signal factor (DSF) were also significantly lowered, exhibiting the inhibition of quorum sensing signaling. However, no noteworthy changes were observed for the phopQ (essential for AvrXA21 activity) and thiG genes (involved with thiazole moiety production in the thiamine biosynthesis pathway) (**Figure 6A**). Overall, niclosamide strongly decreased the transcription of multiple genes involved in Xoo growth and biofilm formation, corroborating its potential as a robust antibacterial drug.

**FIGURE 7:**
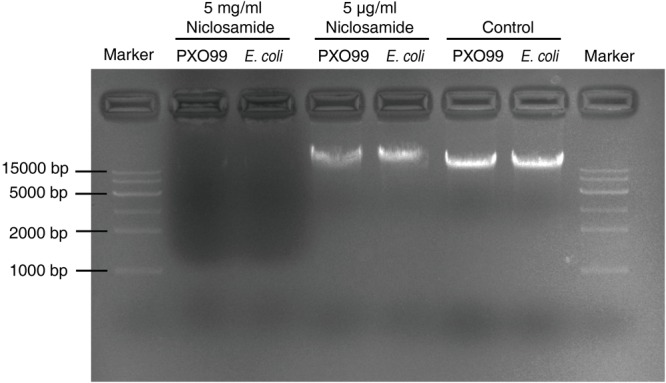
DNA cleavage analysis in response to niclosamide treatment. DNA was extracted from log-phase grown PXO99 and *E. coli*. After quantification the concentration was adjusted to 140 ng/µl, and 5 µl of DNA was mixed with 5 µg/ml or 5 mg/ml of niclosamide and incubated at 28°C for 30 min. The bands were visualized in 1% agarose gel electrophoresis buffered with 1X TAE buffer.

Through Fourier transform infrared spectroscopy (FTIR) study we noticed a drastic shift in the absorbance peaks after niclosamide treatment. The major changes were in the range from 868 cm^-1^ to 3295 cm^-1^. The maximum positive shift was observed at the wavelength of 2843, 1023, and 868 cm^-1^ corresponding to the bonds C–H (aldehyde), C–N (aliphatic amines) and C–H (vinyl) respectively (**Figure 6B** and **Table 2**). The positive shift was also observed for the methylene, CO_2_ and carboxylic acids. However, the maximum negative shift was exhibited by the carboxylic acids (O–H), C–N, and fluoroalkanes (**Figure 6B** and **Table 2**). The spectra clearly showed the striking difference between the control and niclosamide treated samples indicating chemical and physiological changes in Xoo. In addition, we also performed a DNA cleavage analysis which showed that at the lower concentration of 5 µg/ml, niclosamide can partially cause DNA cleavage within 30 min of incubation. However, at 5 mg/ml complete DNA degradation was observed (**Figure 7**).

**Table 2:** FTIR vibrational peak assignment in response to niclosamide treatment.

Vibrational peak assignments		Peak variation		
Bond	Type of bond	Control	Niclosamide	Peak difference
C–H	1,2,4-trisubstituted	868	884	16
C–H	Vinyl	968	971	3
C–N	Aliphatic amines	1023	1041	18
C–X	Fluoroalkanes	1077	1071	–6
C–X	Fluoroalkanes	1119	1117	–2
C–X	Fluoroalkanes	1183	1177	–6
C–O	Carboxylic acids	1259	1262	3
C–O	Carboxylic acids	1289	1292	3
C–H	Methylene	1434	1437	3
C–N	R–N = C = S	1968	1953	–15
O = C = O	Carbon dioxide	2443	2450	7
C–H	Aldehydes	2843	2876	33
C–H	Methylene	2923	2936	13
O–H	Carboxylic acids	3009	2989	–20
N–H	Secondary amines	3109	3116	7
O–H	Alcohols, phenols	3288	3295	7

## Discussion

Although there are several genetic, biological, and chemical control approaches to enhance the rice protection from bacterial leaf blight, the quest for more effective and reliable approach that would provide large-scale protection is still the need of the hour. More than 30 drugs, including antibiotics, have been developed to safeguard crops from pathogen attack (McManus et al., 2002; Xu et al., 2015; Kim et al., 2016a,b; Liang et al., 2016; Rama Devi et al., 2016). Based on multiple experimental approaches like turbidity assay, XTT assay and microtitre plate readings here we showed that niclosamide has a strong inhibitory (>80%) potential against Xoo, and had no detrimental impact on the growth of *E. coli* (**Figure 1**). This non-effectiveness of niclosamide against *E. coli* could be due to the use of lower concentration of niclosamide (5 µg/ml) in this study. In an earlier study, Kim et al. (2016b) also found that niclosamide doesn’t have any significant impact on *E. coli* even at the higher concentration of 50 µg/ml. This observation also implies that niclosamide (at low concentration) can effectively control the growth of Xoo without affecting the growth of other beneficial bacteria. However, to demonstrate this, a large-scale screening is required in the subsequent studies. The antibacterial activity of niclosamide has been also described against *Pseudomonas aeruginosa* (Imperi et al., 2013). Our *in vivo* experiment further demonstrated that niclosamide can effectively block the growth of Xoo thereby impeding the development of bacterial blight disease in rice plants (**Figures 1E,F**). This observation was consistent with the findings of Kim et al. (2016a,b) where they also showed that niclosamide has no harmful effect on the overall vegetative/reproductive growth and yield of the rice plant. In addition, they also showed that niclosamide can induce the expression of defense-related genes including salicylic and jasmonic acid hormones related genes in rice. However, the exact mechanism by which niclosamide inhibits the growth of Xoo still remained unclear.

Microbial biofilms are considered as the most common state of growth for many microbials, and biofilm formation is a key virulence factor for a wide range of microorganisms. Hence, targeting the biofilm formation has emerged as a potential control strategy in the recent years (Yu et al., 2015; Lohse et al., 2017). Therefore, to further understand the inhibitory mechanism of niclosamide, we first tested the efficacy of niclosamide to inhibit the biofilm formation by Xoo. Interestingly, the absence of blue color (after crystal violet staining) indicated the non-adherence of biofilm on tube walls, and clearly displayed the absence of biofilms in niclosamide treated Xoo compared to the controls (**Figures 2A,B**). Biofilms have been extensively studied over the past decades and Confocal Laser Scanning Microscopy (CLSM) has become a standard tool for visualizing biofilms *in situ*, especially to evaluate biofilm architectures and thickness (Tolker-Nielsen and Sternberg, 2014). Therefore, our preliminary observations were further complemented by the confocal microscopic observation of FDA stained Xoo colonies. The considerable reduction in the signal intensity in the niclosamide treated Xoo clearly demonstrated the disruption of biofilm (**Figure 2E**).

Then we further examined the production of xanthomonadins which are the yellow membrane-bound pigments reported to be involved in biofilm formation, and a characteristic feature of xanthomonads (Greek xanthós = yellow) (Poplawsky et al., 2000). The niclosamide drug was found to disrupt the xanthomonadin production (**Figure 2C**) suggesting that niclosamide might directly affect the epiphytic survival of and effective host plant infection. This hypothesis is based on the earlier reports where disruption of xanthomonadin production has been linked to xoo viability and pathogenesis (Park et al., 2009; He et al., 2011). This result motivated us to further test the niclosamide’s ability to disturb EPS production which is yet another well designated crucial factor for plant pathogenesis and the maintenance of cellular integrity (Denny, 1995; Hung et al., 2002). EPS are also considered as an essential component defining the physiochemical properties of a biofilm. Remarkably, the niclosamide drug was found to significantly block the production of EPS, explaining in part that niclosamide can interrupt the growth of pathogenic Xoo, thereby reducing its pathogenicity in rice (**Figure 2D**). Similar observations have been made by several researchers while exploring the efficacy of various chemical compounds such as 1,3,4-oxadiazole moieties (Shi et al., 2015); thiadiazole (Liang et al., 2016) and thymol (Singh et al., 2017). Collectively, our above results suggests that the reduction in the production of xanthomonadin and EPS might be the possible cause of biofilm inhibition by niclosamide.

Our in-depth morphological and ultrastructural analysis by scanning and transmission electron microscopy reveals several noticeable changes in the membrane integrity and overall bacterial morphology after niclosamide treatment. Most antibiotics and antimicrobial peptides have a similar mechanism of action where it causes surface roughening, shrinking, and the formation of potholes, which leads to irreversible bacterial membrane disruption (Hemaiswarya and Doble, 2009; Xu et al., 2010; Shi et al., 2016). Likewise in our study, most bacterial cells showed prevalent membrane damage indicated by the cytoplasm leakage. Though some bacterial cells still maintained the membrane integrity, they were deformed (**Figures 3, 4**). This finding further explains the possible cause of the biofilm inhibition, and strong antibacterial activity by the niclosamide drug. Similar ultra-structural changes have been also reported earlier in response to Ti3C2Tx MXene (Rasool et al., 2016), and difficidin and bacilysin (Wu et al., 2015) on *E coli* and Xoo respectively.

Molecular docking is a key tool in structural molecular biology and computer-assisted drug design which is extensively being used to predict the predominant binding mode(s) of a ligand with a protein of known three-dimensional structure (Morris and Lim-Wilby, 2008). Our results shed light on the probable molecular interaction between niclosamide with all the tested proteins, which is quite evident by the binding affinity score of > -8 Kcal/mol compared to the positive controls bismerthiazol and probenazole (**Figure 5** and **Table 1**). This docking score is in accordance with earlier reports signifying that lower the binding affinity score, higher will be the binding efficiency (Sahu et al., 2012; Singh et al., 2017). In all the interactions there were involvements of at least two to three hydrogen bonds within the predicted active site (**Figure 5**). This existence of intra/intermolecular hydrogen bonding between the ligand (niclosamide) and the target (protein) further demonstrate strong binding interaction (Musthafa et al., 2013; Singh et al., 2017). Thiazole, isothiazole, thiadiazole and their derivatives are reported to induce host defenses against plant pathogens. Particularly, probenazole and bismerthiazol are proven chemicals to control the bacterial leaf blight disease in rice (Shi et al., 2015; Liang et al., 2016; Yu et al., 2016). Recently, by employing similar docking approach, Singh et al. (2017) studied the inhibitory action of individual components of thyme oil on DSF synthase protein.

Taking into account the strong binding affinity cum molecular interaction of niclosamide with several proteins, we step forwarded to confirm these results by experimental analysis. Corroborating our above results and findings, the quantitative gene expression analysis of gum, xanthan and EPS-related genes showed significant downregulation in response to niclosamide treatment (**Figure 6A**). Substantiating our experimental results, XanA showed the highest level (5.5 fold) of reduction. XanA is known to encode phosphoglucomutase/phosphomannomutase which converts glucose-6-phosphate into glucose-1-phosphate in the first step of EPS biosynthesis (Hung et al., 2002). It is also one of the most important virulence factors of Xanthomonads (Jansson et al., 1975). Moreover, the EPS biosynthesis related genes belonging to the gum gene cluster were also found to significantly reduce. Most importantly, the rpfB gene responsible for the production of diffusible signal factor (DSF) was also significantly affected. After screening a library of FDA-approved chemicals, Imperi et al. (2013) also described that niclosamide targets some regulatory pathway(s) responsive to the energetic/metabolic status of the cell which is ultimately required for full activity of the QS signaling network in *P. aeruginosa*. As gumB, gumD, gumG, and gumM belongs to the same gene cluster which is responsible for EPS biosynthesis, there might be a common regulon subject to positive feedback amplification which controls the transcription of these genes (Vojnov et al., 1998, 2001). Therefore, it is very likely that niclosamide damp the transcription of these genes. A similar observation has been recently reported in Xoo by Singh et al. (2017) where they found reduced expression of genes associated with motility, EPS and virulence in response to thyme oil treatment. In order to develop more specific niclosamide-based quorum sensing inhibitors, bioinformatics and experimental studies could be pursued in future by introducing random chemical modifications in the salicylanilide structure.

Niclosamide has been recommended as a safer and environmental friendly drug by FDA. Niclosamide degraded rapidly in the pond and river sediments incubated under aerobic, static conditions with half-lives of 1.1 and 3.9 days, respectively (Muir and Yarechewski, 1982; Ofori-Adjei et al., 2008). Finally, by utilizing the Fourier transform infrared spectroscopy (FTIR) we found noteworthy changes in the absorbance peaks after niclosamide treatment. The results revealed major positive shifts in C–H (aldehyde), C–N (aliphatic amines), and C–H (vinyl). While, the maximum negative shift was exhibited by the carboxylic acids (O–H), C–N, and fluoroalkanes (**Figure 6B**). This finding is in accordance with that of a previous study on the ciprofloxacin penetration in *P. aeruginosa* biofilm (Suci et al., 1994). They demonstrated significant changes in the IR bands associated with proteins, nucleic acids, and carbohydrates following exposure to the antibiotic. Similarly, in yet another recent study, Yahya et al. (2017) studied the antibiofilm activity and mode of action of DMSO alone and its combination with afatinib against Gram-negative pathogens and observed significant changes in the IR spectra of proteins (1700–1500 cm^-1^), nucleic acids, and polysaccharides (1300–900 cm^-1^) in EPS matrix. Overall, our FTIR analysis demonstrated the substantial damage caused by niclosamide at the chemical level. This result was further substantiated by DNA cleavage assay where niclosamide was found to degrade the DNA suggesting the possibility of inhibition of intracellular functions via interference with DNA/RNA functions (Shi et al., 2016).

## Conclusion

**FIGURE 8:**
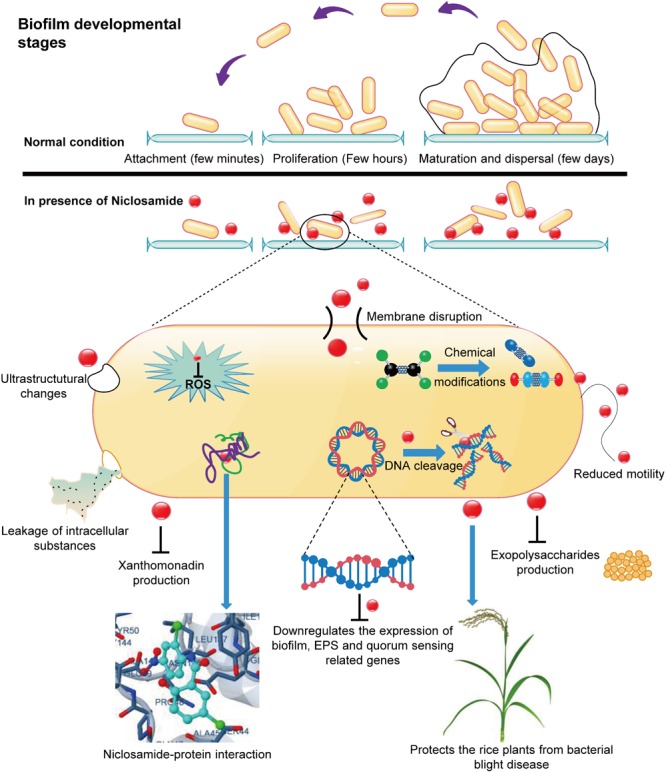
The proposed model depicting the mechanism of Xoo’s biofilm disruption by niclosamide drug and its utility to protect rice plants from highly destructive bacterial blight disease caused by Xoo. The spherical red dot denotes the niclosamide drug. (This illustrative model was drawn in Edraw Max software, Version 9.0).

To conclude, we summarized the whole work in an illustrative model depicting the mechanism of action of niclosamide against Xoo (**Figure 8**). Together with the earlier reports and our present findings we confirmed that niclosamide can impede the biofilm formation by disrupting the membrane permeability, and significantly affect the xanthomonadin, EPS synthesis and quorum sensing signaling in Xoo, and parallelly induce considerable chemical modifications. Moreover, our analyses showed promising results demonstrating the applicability of niclosamide as a safer, effective and environmental friendly drug to control the detrimental bacterial blight disease in rice plants. Consistent with the earlier presumptions here we substantiated niclosamide as a master regulatory drug that effectively functions to protect both animals and plants from contagious diseases caused by parasites and pathogenic bacteria respectively. However, by taking into account the multiple impacts of niclosamide on Xoo, the involvement of other inhibitory mechanisms cannot be ruled out. Further research on transcriptome level changes might reveal more detailed mechanistic insights.

## Author Contributions

SKS and NY: conceived and designed the experiments and wrote the manuscript. SKS and PZ: performed the experiments and analyzed the data. All the authors read and approved the final manuscript.

## Conflict of Interest Statement

The authors declare that the research was conducted in the absence of any commercial or financial relationships that could be construed as a potential conflict of interest.

## Footnotes

1 www.acdlabs.com

## Supplementary Material

FIGURE S1 The 2D molecular interaction of bismerthiazol and probenazole with the biofilm and quorum sensing related proteins as visualized in Discovery studio software.

TABLE S1 List of primers used for gene expression analysis in this study.
