# Supplementary material for: Niclosamide Blocks Rice Leaf Blight by Inhibiting Biofilm Formation of Xanthomonas oryzae
Source: Front Plant Sci. 2018 Mar 29;9:408. doi: 10.3389/fpls.2018.00408 (PMC5884940; doi:10.3389/fpls.2018.00408)
Supplement: FIGURE S1 — The 2D molecular interaction of bismerthiazol and probenazole with the biofilm and quorum sensing related proteins as visualized in Discovery studio software. [file Table_1.DOC]

**Table S1.** List of primers used for gene expression analysis in this study

| **Primer name** | **Sequence (5'-3')** |
| --- | --- |
| thiC-F | CTCTGCTATGTCACTCCGAAAG |
| thiC-R | GCGATCTTGTAGGCCATGAT |
| thiE-F | GGTTGCAATACCGCAACAAG |
| thiE-R | ATGATCAATGGCACACCGT |
| gumB-F | ATCGTGTTCCGCATGGTC |
| gumB-R | ATATCGCCGCCGTAAATCTC |
| gumD-F | GCGTGCGATTCGTATGTTTC |
| gumD-R | GCGAGGAAGCTGTGTAAGT |
| gumG-F | GCCAATGGTTCACGTCTGTA |
| gumG-R | GCACGCAAGGCAATGTAAG |
| gumM-F | TGTTGAGACGACGGGAATTG |
| gumM-R | CTGCCAACGCATGGAATAGA |
| rpfG-F | GACGAGATGAGCGTGATGAA |
| rpfG-R | GATAACCGGTGCCGTCATAA |
| rpfB-F | ACGCGGTGAAGCGGGTCTTT |
| rpfB-R | ATCACTGCGCTGCCGCTGTA |
| hutU-F | ACGGGCCAATACCGCGACAA |
| hutU-R | ACATCCGCGCCATGCTCACT |
| hutG-F | ATGTTCGGGGTGCGCGTAGT |
| hutG-R | TCCGCTCGTTCATCCCGCAT |
| PhoPQ-F | TGCCAGCGGCGACGATTTCT |
| PhoPQ-R | CGGATGTTGTGCGCGCTGTT |
| impA-F | CGCGGCTTTTTCGGCCAAGT |
| impA-R | ACCACCCGCAAGGAAGCGTT |
| xanA-F | TCGTGGAAGACGCGCACGAA |
| xanA-R | AAGCCGCTCAAGCTGGTGGT |
| gyrB-F | CCTGTTGCTGACCTTCTTCTAC |
| gyrB-R | CTTCAGATACAGCTCGCTCTTG |
