# Supplementary figures and images for: Niclosamide Blocks Rice Leaf Blight by Inhibiting Biofilm Formation of Xanthomonas oryzae
Source: Front Plant Sci. 2018 Mar 29;9:408. doi: 10.3389/fpls.2018.00408 (PMC5884940; doi:10.3389/fpls.2018.00408)

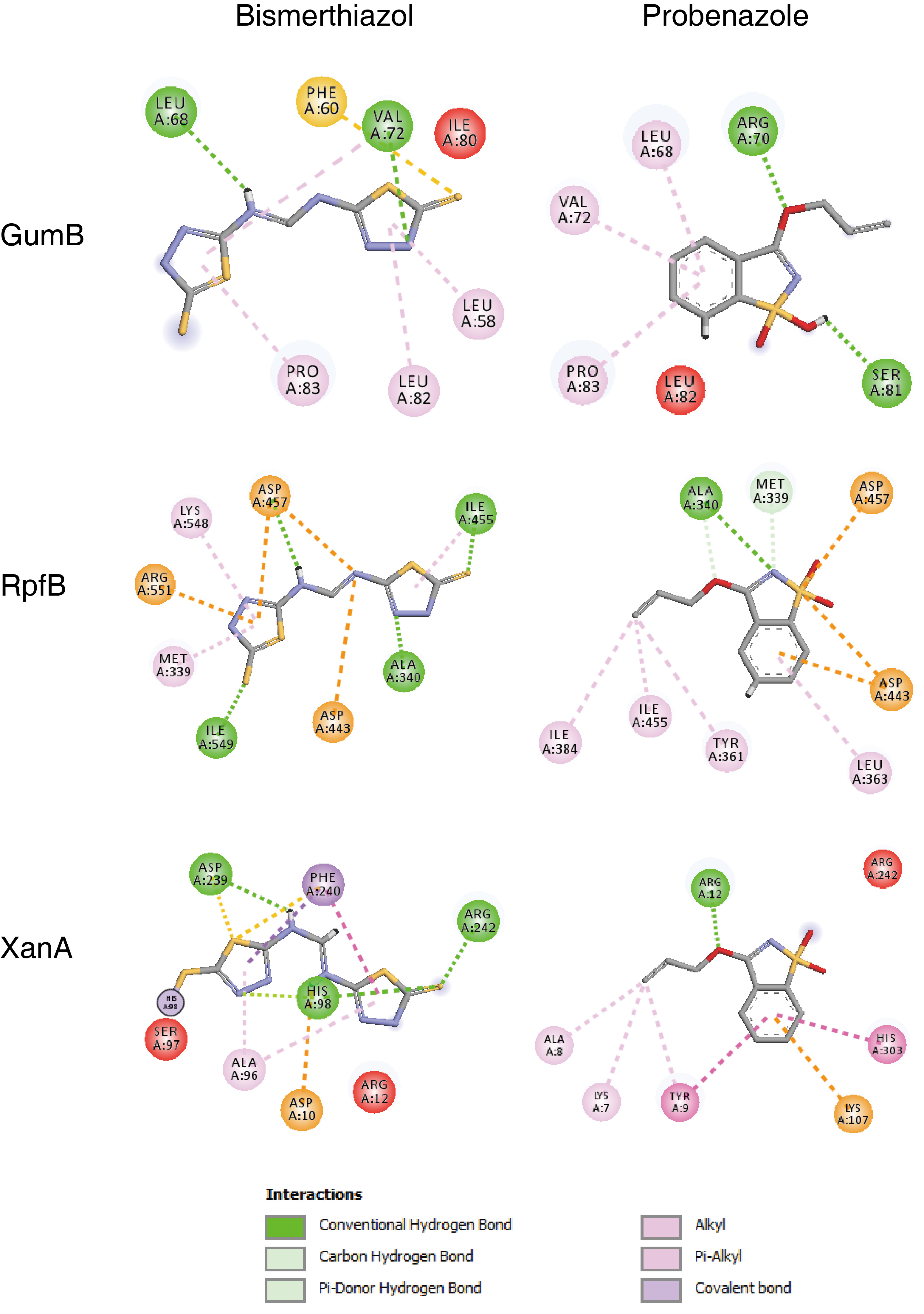

Supplement: TABLE S1 — List of primers used for gene expression analysis in this study. [file Image_1.JPEG]
